# Associations of melatonin receptor gene polymorphisms with Graves' disease

**DOI:** 10.1371/journal.pone.0185529

**Published:** 2017-09-29

**Authors:** Jiunn-Diann Lin, Shun-Fa Yang, Yuan-Hung Wang, Wen-Fang Fang, Ying-Chin Lin, Bing-Chun Liou, Yuh-Feng Lin, Kam-Tsun Tang, Chao-Wen Cheng

**Affiliations:** 1 Graduate Institute of Clinical Medicine, College of Medicine, Taipei Medical University, Taipei, Taiwan; 2 Division of Endocrinology, Department of Internal Medicine, Shuang-Ho Hospital, Taipei Medical University, New Taipei City, Taiwan; 3 Division of Endocrinology and Metabolism, Department of Internal Medicine, School of Medicine, College of Medicine, Taipei Medical University, Taipei, Taiwan; 4 Institute of Medicine, Chung Shan Medical University, Taichung, Taiwan; 5 Department of Medical Research, Chung Shan Medical University Hospital, Taichung, Taiwan; 6 Department of Medical Research, Shuang-Ho Hospital, Taipei Medical University, New Taipei City, Taiwan; 7 Department of Family Medicine, Shuang-Ho Hospital, Taipei Medical University, New Taipei City, Taiwan; 8 Division of Nephrology, Department of Internal Medicine, Shuang-Ho Hospital, Taipei Medical University, New Taipei City, Taiwan; 9 Division of Endocrinology and Metabolism, Department of Internal Medicine Taipei Veterans General Hospital, Taipei, Taiwan; German Cancer Research Center (DKFZ), GERMANY

## Abstract

**Background:**

Melatonin plays an important role in immunity and has been linked to autoimmune diseases. Possible associations of single-nucleotide polymorphisms (SNPs) of melatonin receptor type 1A (MTNR1A) and 1B (MTNR1B), with autoimmune thyroid disease in an ethnic Chinese (i.e., Taiwanese) population were examined.

**Materials and methods:**

Totally, 83 Hashimoto’s thyroiditis patients, 319 Graves’ disease (GD), and 369 controls were recruited. Three SNPs (rs6553010, rs13140012, and rs2119882) of MTNR1A and three SNPs (rs1387153, rs10830963, and rs1562444) of MTNR1B were genotyped.

**Results:**

There were a reduced frequency of the C allele of rs2119882 and a reduced percentage of the CC+CT genotype in the GD group compared to the control group (*p* = 0.039, odds ratio (OR) = 0.79, 95% confidence interval (CI) = 0.63~0.99, and *p* = 0.032, OR = 0.72, 95% CI = 0.53~0.97, respectively). There was a significant difference in the percentage of the AT haplotype of the combination of rs13140012 and rs2119882 between the GD and control groups (*p* = 0.010, OR = 1.34, 95% CI = 1.07~1.67). In addition, there were significant associations of anti-thyroid peroxidase antibody titers with rs13140012 and rs2119882, and the AATT genotype of the combination of rs13140012 and rs2119882 (*p* = 0.003, 0.003, and 0.004, respectively). There were no significant associations of SNPs and possible haplotypes of MTNR1B with susceptibility to GD.

**Conclusions:**

Genetic variants of rs2119882 of MTNR1A and the AT haplotype of the combination of rs2119882 and rs13140012 were associated with GD susceptibility in an ethnic Chinese population. The results support the involvement of the melatonin pathway in the pathogenesis of GD.

## Introduction

Melatonin exerts multiple biological functions, including promoting sleep, regulating circadian and seasonal rhythms, promoting anti-aging, antioxidation, anti-tumorigenesis, and anti-apoptosis, eliminating free radicals, and controlling the onset of puberty [1,2,3,4]. In addition, evidence shows that melatonin plays a critical role in regulating both innate and adaptive immune responses and the balance of T-helper 1/T-helper 2 cytokines [5,6]. Melatonin deprivation and exogenous melatonin administration can induce immune dysregulation [7,8]. Moreover, melatonin is even considered to be the third signal triggering an immune response together with the human leukocyte antigen-processed antigen-T cell receptor and costimulatory molecule expressions [9]. Because of its strong immunoregulatory function, melatonin has been linked to several autoimmune diseases, including systemic lupus erythematosus, multiple sclerosis, type 1 diabetes mellitus, rheumatoid arthritis, and inflammatory bowel disease [10,11].

In humans, two major types of melatonin receptor (MTNR) genes, the MTNR1A gene, encoding MT1, and the MTNR1B gene, encoding MT2, were identified, and their genetic variants were linked to several diseases [12,13]. Genetic variants of MTNR1A were linked to tumor formation, coronary artery disease, scoliosis, etc. [14,15,16]. On the other hand, single-nucleotide polymorphisms (SNPs) of MTNR1B were reported to be associated with glucose intolerance, cardiovascular diseases, systemic lupus erythematosus, and rheumatoid arthritis [17,18]. It was also reported that melatonin can bind to MTNRs of immunocytes, control the downstream intracellular signaling pathway, and subsequently directly modulate immune reactions [19,20].

The potential association between melatonin and thyroid functions was described in previous studies. Lewinski et al. showed that melatonin can repress mitosis of thyroid follicular cells in vivo and in a cell culture system [21]. Wright et al. indicated that the administration of melatonin directly suppressed thyroid hormone secretion [22,23]. However, Gordon et al. demonstrated that there was no significant change in circulating thyroid hormone levels but an increased total thyroxine (T4) content in the thyroid gland in male weanling rats with chronic melatonin intake compared to controls [24]. Interestingly, recent evidence showed that melatonin, its key enzymes required for melatonin synthesis, and the MT1 receptor protein are present in the thyroid gland, which suggests that melatonin can also influence thyroid-specific gene expressions, and thyroid hormone synthesis and regulation [25].

Autoimmune thyroid disease (AITD) consists of a spectrum from Graves' disease (GD) with high thyroid function to Hashimoto's thyroiditis (HT) with declined thyroid function. GD is mainly driven by humoral immunity, which results in the formation of a pathogenic thyroid-stimulating hormone (TSH) receptor antibody (TSHRAb). The TSHRAb binds to TSH receptors of thyroid follicular cells and subsequently stimulates hyperplasia in thyroid follicular cells, enhances thyroid-specific gene expressions, and increases thyroid hormone synthesis. On the other hand, HT is mainly derived from cell-mediated immune reactions, which induce thyroid follicular cell apoptosis, increase lymphocyte infiltration into the thyroid gland, destroy thyroid follicles, and finally reduce thyroid function [26].

It is well established that AITD results from interactions of genetic susceptibility and environmental factors, which lead to a breakdown in immune tolerance [26]. Tang et al. suggested that melatonin can alleviate oxidative stress and reduce DNA damage of lymphocytes of GD patients in vitro [27]. In our previous study, we found a possible association of genetic variants of B-cell-activating factor with the occurrences of GD and AITD, and production levels of an autoantibody [28]. In addition, melatonin was reported to be able to offset BAFF overexpression in cells of systemic lupus erythematosus patient [29]. This evidence further implies potential regulatory mechanisms of melatonin on autoantibody production. As the melatonin-MTNR signaling pathway is associated with immune regulation, autoimmune diseases, and the thyroid gland itself, it is not surprising that the melatonin-MTNR signal may also play certain roles in AITDs. However, no study has yet been conducted to investigate the impact of the melatonin-MTNR pathway on AITDs. In this study, we further explored possible associations of SNPs of MTNR1A and MTNR1B with AITDs in an ethnic Chinese (i.e., Taiwanese) population.

## Materials and methods

### Subjects

As in our previously published article [28], blood specimens were obtained by the Division of Endocrinology, Shuang-Ho Hospital from January 2013 to September 2014, including 319 patients with GD and 83 patients with HT. In addition, 369 blood samples of subjects without AITD or other autoimmune diseases were collected from the Health Screening Center of Shuang-Ho Hospital in May to August 2014. The study protocol was approved by the Taipei Medical University-Joint Institutional Review Board. Written informed consent was obtained from all subjects before participation. All enrolled participants were older than 20 years. Pregnant women and subjects with a history of intoxication or alcoholism were excluded. GD and HT were determined according to the criteria as previously described [28].

### Genotyping

SNPs were differentiated using the Taqman assay and were subsequently determined by Sequence Detection System vers. 3.0 software (Applied Biosystems, Foster City, CA). A real-time polymerase chain reaction was performed by adding 10 ng of DNA to the buffer, containing 5 μL of MasterMix, 0.25 μL of a probe, and 1 μL each of the forward and reverse primers, in a total volume of 10 μL. The mixtures were amplified in the Applied Biosystems Step-one Real-time Polymerase Chain Reaction system according to manufacturer’s protocol. Information on the SNPs is given in Table A in S1 File.

### Laboratory analysis

Serum free thyroxine (FT4), TSH, anti-thyroid peroxidase antibody (anti-TPO Ab), and anti-thyroglobulin antibody (ATA) titers were measured as previously described [30].

### Statistical analysis

SPSS vers. 13.0 for Windows (SPSS, Chicago, IL) was used for all statistical analyses. Quantitative values are shown as the mean ± standard deviation (SD). An independent *t*-test was used to compare differences in demographic data, FT4, TSH, and TSHRAb between the two groups. A χ^2^ test was used to assess differences in the GD, HT, or AITD groups with the control group. Comparisons of clinical parameters among the GD, HT, and control groups were performed by a one-way analysis of variance analysis with Bonferroni’s post-hoc tests. A test of Hardy-Weinberg equilibrium was performed with the χ^2^ test. The χ^2^ test or Fisher’s exact test was also used to assess differences in categorical data between the two groups.

We initially assessed associations of the susceptibility to GD, HT, or AITD with demographic parameters and SNPs using a univariate logistic regression. Significant distinguishing factors were determined and were further analyzed by a multivariate logistic regression. Pairwise linkage disequilibrium and associations of haplotypes with susceptibility to GD, HT, and AITD were investigated using Haploview software vers. 4.2. All statistical tests were two-sided, and a *p* value of < 0.05 was considered significant.

## Results

### Demographic data of the GD, HT, AITD, and control groups

Table B in S1 File presents demographic data of the enrolled HT, GD, AITD, and control groups. Patients with HT were older than the healthy controls. Meanwhile, a higher prevalence of females was observed in the HT than in the GD and control groups. A higher percentage of patients with GD had a smoking habit than in the control group. Patients in both the GD and HT groups had higher prevalences of a family history of thyroid disease than did patients in the control group.

### Associations of SNPs of MTNR1A and MTNR1B with susceptibility to AITD

**Fig 1:**
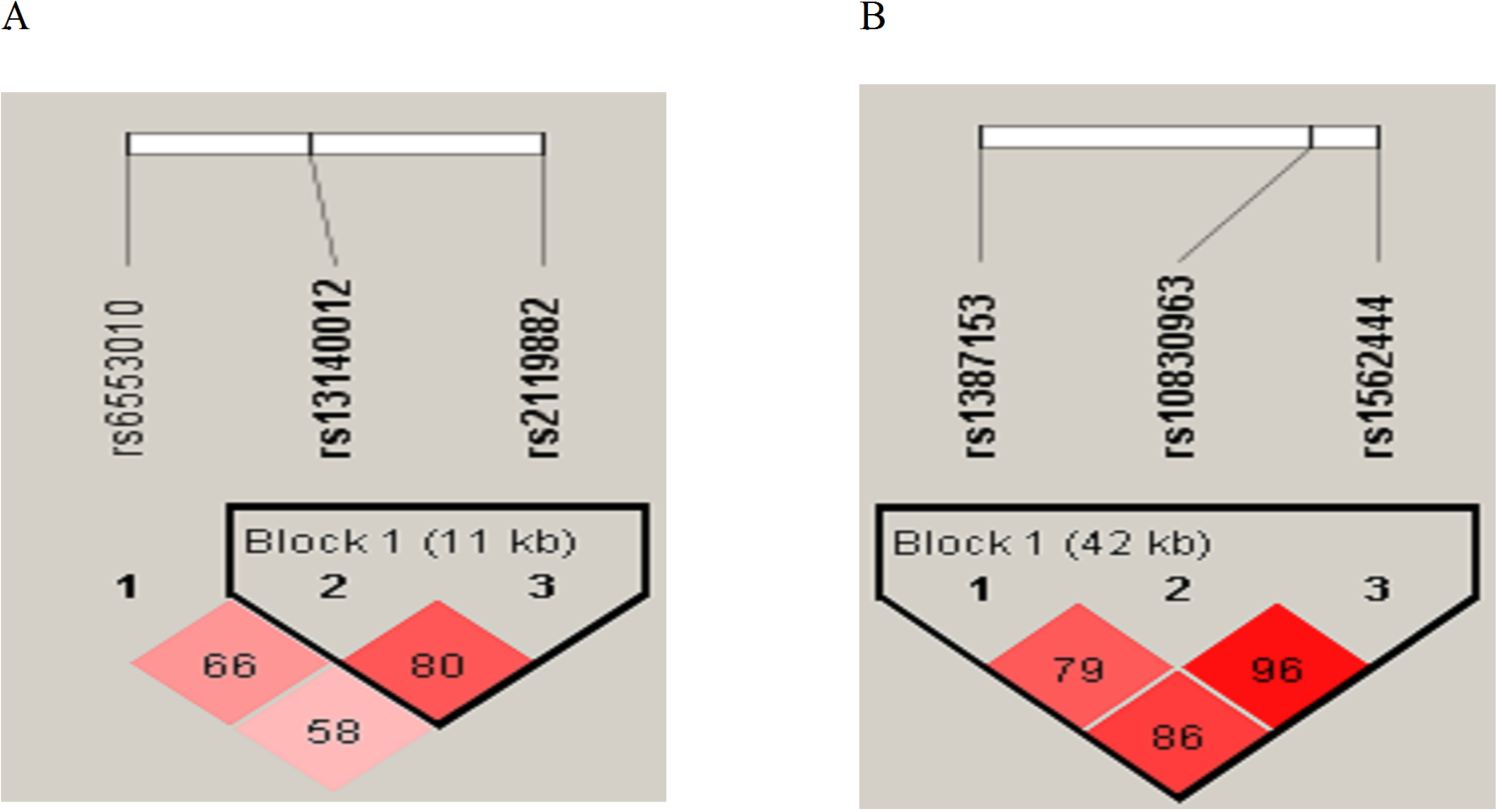
Pairwise linkage disequilibrium patterns of melatonin receptor type 1A (panel A) and type 1B (panel B).

The linkage disequilibrium blocks of three SNPs of the MTNR1A gene and three SNPs of the MTNR1B gene are shown in Fig 1A and 1B, respectively. Genotype frequencies of the three SNPs of MTNR1A (rs6553010, rs13140012, and rs2119882) and the three SNPs of MTNR1B (rs1387153, rs10830963, and rs1562444) were determined in all groups (Tables 1 and 2), and all of them exhibited Hardy-Weinberg equilibrium (data not shown). As MTNR1B SNPs have been linked to glucose intolerance, subjects without hyperglycemia (GD = 15, HT = 1, healthy controls = 60) and all subjects without excluding glucose intolerance were both analyzed in the MTNR1B association study. Differences in genotypic and allelic frequencies of the SNPs of MTNR1A in the GD, HT, AITD, and control groups were compared, and results are shown in Table 1. There were significantly lower frequencies of the C allele of rs2119882 in the GD group than in the control group (Table 1). Meanwhile, there was a reduced percentage of the CC+CT genotype in the GD (*p* = 0.032) group compared to the control group (Table 1). There was no significant difference in the frequency of the T allele of rs13140012 between the GD and control groups (Table 1, *p* = 0.062). Besides, there was no significant difference in the percentage of the AT+TT genotype in rs13140012 between the GD (*p* = 0.069) and control groups. There were no significant differences in allelic or genotypic frequencies of rs6553010 between GD and control groups (Table 1). In addition, there were no significant differences in allelic or genotypic percentages of all three SNPs of MTNR1B between the GD and control groups, between the HT and control groups, or between the AITD and control groups whether analyzing only those without glucose intolerance or all participants (Table 2 and Table C in S1 File, respectively). Finally, to determine differences in genetic effects on the susceptibility to GD in different genders, we respectively evaluated the association of genetic variants of MTNRA with GD in females and males, and results are shown in Table D in S1 File. There were no associations of the three MTNR1A SNPs with GD in females, while there were significant decreases in the T allele and TT genotype in rs13140012 in the GD group compared to the control group in males (*p* = 0.044 and 0.044, respectively, Table D in S1 File).

**Table 1:** Genotypic and allelic frequencies of rs13140012, rs6553010, and rs2119882 in the melatonin receptor type 1A gene.

Polymorphism	Control*n* (%)	GD*n* (%)	HT*n* (%)	AITD*n* (%)	OR1 (95% CI)	OR2 (95% CI)	OR3 (95% CI)
rs6553010							
AA	159 (45.3)	162 (50.9)	30 (36.1)	192 (47.8)	1	1	1
AG	149 (42.5)	120 (31.7)	39 (47.0)	159 (39.7)	0.79 (0.57~1.09)	1.39 (0.82~2.35)	0.83 (0.65~1.20)
GG	43 (12.3)	36 (11.3)	14 (16.9)	50 (12.5)	0.82 (0.50~1.35)	2.34 (0.24~22.7)	0.96 (0.61~1.52)
AG+GG	192 (54.8)	156 (43.0)	53 (63.9)	209 (52.2)	0.80 (0.59~1.08)	1.46 (0.89~2.40)	0.90 (0.68~1.20)
Allele							
A	467 (66.5)	444 (69.8)	99 (60.7)	543 (67.7)	1	1	1
G	235 (33.5)	192 (30.2)	67 (39.3)	259 (32.3)	0.86 (0.68~1.08)	1.35 (0.95~1.90)	0.95 (0.76~1.18)
rs13140012							
AA	140 (39.9)	149 (46.9)	26 (31.3)	175 (43.6)	1	1	1
AT	165 (47.0)	136 (42.8)	46 (55.4)	182 (45.4)	0.77 (0.56~1.07)	1.50 (0.88~2.55)	0.88 (0.65~1.20)
TT	46 (13.1)	33 (10.4)	11 (13.3)	44 (11.0)	0.67 (0.41~1.12)	1.29 (0.59~1.81)	0.73 (0.46~1.17)
AT+TT	201 (60.1)	169 (53.2)	57 (68.7)	216 (56.4)	0.75 (0.55~1.02)	1.46 (0.87~2.42)	0.85 (0.64~1.14)
Allele							
A	445 (63.4)	434 (68.2)	98 (59.0)	532 (66.3)	1	1	1
T	257 (36.6)	202 (31.8)	68 (41.0)	270 (33.7)	0.81 (0.64~1.01)	1.20 (0.85~1.70)	0.87 (0.70~1.07)
rs2119882							
TT	140 (39.9)	153 (48.1)	28 (33.7)	181 (45.1)	1	1	1
CT	166 (47.3)	132 (41.5)	43 (51.8)	175 (43.6)	0.73 (0.53~1.01)	1.30 (0.77~2.19)	0.82 (0.60~1.11)
CC	45 (12.8)	33 (10.4)	12 (14.5)	45 (11.2)	0.67 (0.41~1.11)	1.33 (0.63~2.84)	0.77 (0.48~1.24)
CC+CT	211 (60.1)	165 (51.9)	55 (66.3)	220 (54.8)	0.72(0.53~0.97*	1.30 (0.79~2.76)	0.81 (0.60~1.08)
Allele							
T	446 (63.5)	438 (68.9)	99 (60.7)	537 (67.0)	1	1	1
C	256 (36.5)	198 (31.1)	67 (39.3)	265 (33.0)	0.79(0.63~0.99*	1.18 (0.83~1.67)	0.86 (0.70~1.06)

GD, Graves’ disease; HT, Hashimoto’s thyroiditis; AITD, autoimmune thyroid disease (GD + HT); control, control group. OR1, odds ratio 1, GD vs. the control; OR2, odds ratio 2, HT vs. the control; OR3, odds ratio 3, AITD vs. the control; CI, confidence interval

* *p*<0.05.

**Table 2:** Genotypic and allelic frequencies of rs1387153, rs10830963, and rs1562444 in the melatonin receptor type 1B gene.

Polymorphism	Control*n* (%)	GD*n* (%)	HT*n* (%)	AITD*n* (%)	OR1 (95% CI)	OR2 (95% CI)	OR3 (95% CI)
rs1387153							
CC	101 (33.8)	93 (30.7)	27 (32.9)	120 (31.2)	1	1	1
CT	137 (45.8)	149 (49.2)	43 (52.4)	192 (49.9)	1.18 (0.82~1.70)	1.17 (0.68~2.03)	1.18 (0.84~1.66)
TT	61 (20.4)	61 (20.1)	12 (14.6)	73 (18.9)	1.09 (0.69~1.71)	0.74 (0.35~1.56)	1.01 (0.66~1.55)
CT+TT	198 (66.2)	156 (69.3)	55 (67.0)	265 (68.8)	1.15 (0.82~1.62)	1.04 (0.62~1.75)	1.13 (0.82~1.56)
Allele							
C	339 (56.7)	335 (55.3)	97 (59.1)	432 (56.1)	1	1	1
T	259 (43.3)	271 (44.7)	67 (40.9)	338 (43.9)	1.06 (0.84~1.33)	0.90 (0.64~1.28)	1.02 (0.83~1.27)
rs10830963							
CC	96 (32.1)	91 (30.0)	25 (30.4)	116 (30.1)	1	1	1
CG	147 (49.2)	143 (47.2)	44 (53.7)	187 (48.6)	1.03 (0.71~1.48)	1.15 (0.66~2.00)	1.05 (0.75~1.49)
GG	56 (18.7)	69 (22.8)	13 (15.9)	82 (21.3)	1.30 (0.83~2.05)	0.89 (0.42~1.88)	1.21 (0.79~1.87)
CG+GG	203 (77.9)	169 (70.0)	57 (69.6)	269 (69.9)	1.10 (0.78~1.56)	1.08 (0.64~1.83)	1.10 (0.79~1.52)
Allele							
C	339 (56.7)	325 (53.6)	94 (57.3)	419 (54.4)	1	1	1
G	259 (43.3)	281 (46.4)	70 (42.7)	351 (45.6)	1.13 (0.90~1.42)	0.98 (0.69~1.38)	1.10 (0.88~1.36)
rs1562444							
AA	133 (44.5)	139 (45.9)	38 (46.3)	177 (46.0)	1	1	1
AG	132 (44.1)	131 (43.2)	40 (48.8)	171 (44.4)	0.95 (0.68~1.33)	1.06 (0.64~1.76)	0.97 (0.71~1.34)
GG	34 (11.4)	33 (10.9)	4 (4.9)	37 (9.6)	0.93 (0.54~1.59)	0.41 (0.14~1.23)	0.82 (0.49~1.37)
AG+GG	166 (55.5)	164 (54.1)	44 (53.7)	208 (54.0)	0.95 (0.69~1.30)	0.93 (0.57~1.52)	0.94 (0.70~1.28)
Allele							
A	398 (66.6)	409 (67.5)	116 (70.7)	525 (68.2)	1	1	1
G	200 (33.4)	197 (32.5)	48 (29.3)	245 (31.8)	0.96 (0.75~1.22)	0.82 (0.57~1.30)	0.93 (0.74~1.17)

GD, Graves’ disease; HT, Hashimoto’s thyroiditis; AITD, autoimmune thyroid disease (GD + HT); control, control group. OR1, odds ratio 1, GD vs. the control; OR2, odds ratio 2, HT vs. the control; OR3, odds ratio 3, AITD vs. the control; CI, confidence interval.

### Associations of haplotypes of MTNR1A and MTNR1B with the GD, HT, AITD, and control groups

We further investigated possible haplotypes of rs13140012, rs6553010, and rs2119882 of MTNR1A for susceptibility to GD, HT, and AITD. We initially analyzed haplotypes of the combination of rs6553010, rs13140012, and rs2119882 of MTNR1A, and results are shown in Table E in S1 File. We found that the CTA haplotypes of rs2119882, s13140012, and rs6553010 reported by Lin et al., which are associated with susceptibility to oral cancer, did not contribute to susceptibility to GD [31]. However, due to the relatively low D’ value between rs6553010 and rs2119882 (D’ = 0.58 < 0.7) [32], we mainly focused on the rs13140012 and rs2119882 haplotypes (D’ = 0.80) in the current study (Fig 1A and Table 3). In MTNR1A, rs13140012 and rs2119882 exhibited high linkage disequilibrium in the GD and control groups (D' = 0.80). The most common haplotype was AT, followed by TC, TT, and AC in the GD, HT, AITD, and control groups. There was a significant difference in the AT frequency between the GD and control groups (*p* = 0.010). These findings suggest that the AT haplotypes of rs13140012 and rs2119882 were associated with susceptibility to GD. On the contrary, no significant association of TC, TT, or AC haplotypes with the GD, HT, or AITD groups was observed (Table 3). Possible haplotypes of rs1387153, rs10830963, and rs1562444 of MTNR1B were also explored, and results are shown in Table F in S1 File. The most prevalent haplotype was TGA, followed by CCG, CCA, and CGA. There were no significant differences in the frequencies of these haplotypes between the GD and control groups, between the HT and control groups, or between the AITD and control groups.

**Table 3:** Combined haplotype frequencies of rs13140012 and rs2119882 in Graves’ disease (GD) and in controls.

Total	GD (318)	Control (351)	OR (95% CI)	*p* value
AT	0.649	0.580	1.34 (1.07~1.67)	0.010
TC	0.278	0.311	0.85 (0.67~1.08)	0.182
TT	0.040	0.055	0.68 (0.40~1.13)	0.194
AC	0.033	0.053	0.60 (0.35~1.03)	0.074

OR, odds ratio; CI, confidence interval.

### Associations of SNPs of MTNR1A and MTNR1B with thyroid function, and TSHRAb and anti-TPO Ab titers at the baseline in the GD, HT, and AITD groups

There were no significant associations of the severity of GD or HT with these six SNPs (data shown in Table G in S1 File). TSHRAb titers at the baseline in patients also showed no significant association in the GD group with genotypes of rs6553010, rs13140012, or rs2119882 of MTNR1A (*p* = 0.357, 0.295, and 0.160, Fig A1, A2 and A3 in S1 File, respectively). At the same time, there was no significant difference in baseline TSHRAb levels between the AATT and non-AATT genotypes of rs13140012 and rs2119882 (*p* = 0.302, Fig A4 in S1 File). Similarly, there was no association of TSHRAb levels with genotypes of the three SNPs and their combined genotypes of MTNR1B (data not shown).

**Fig 2:**
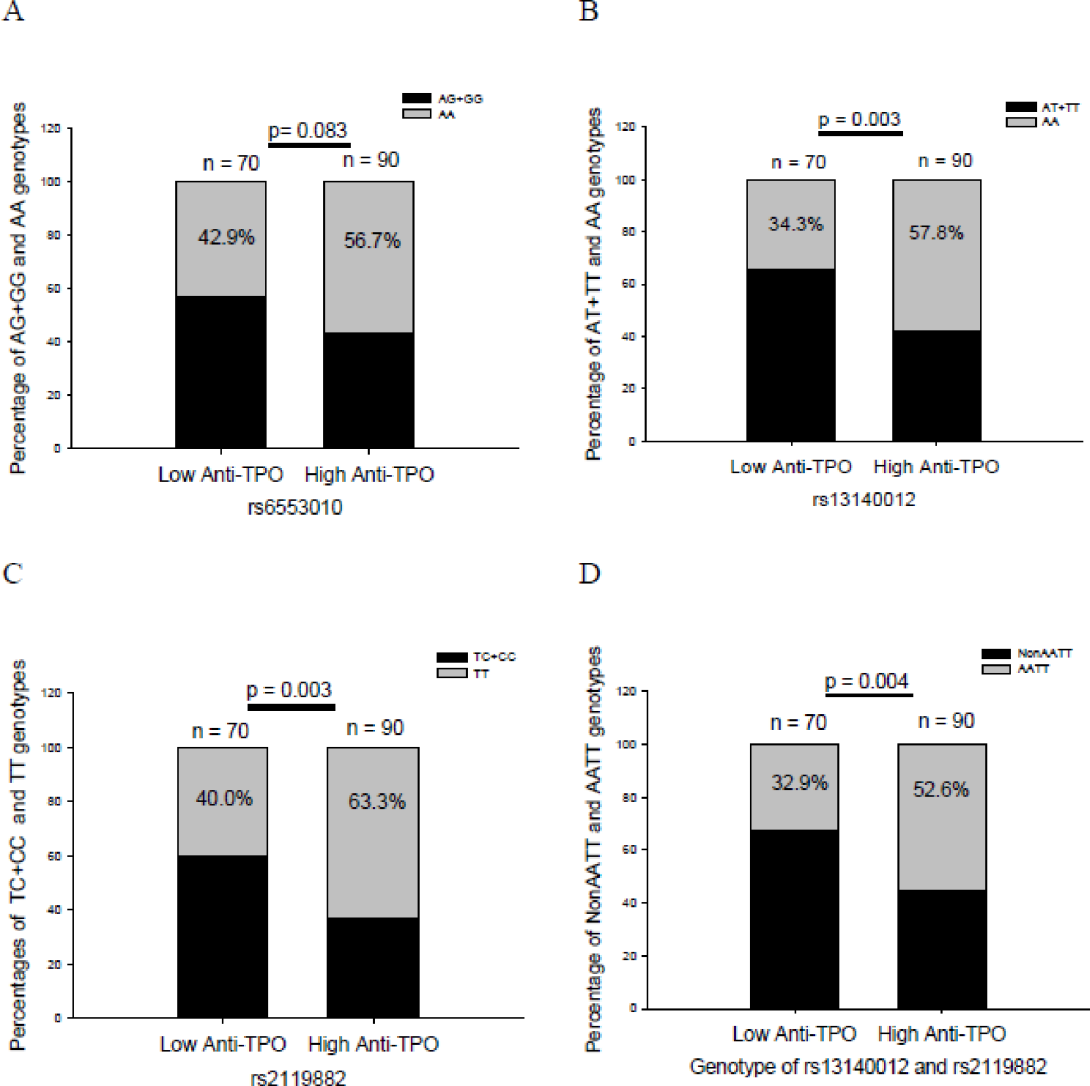
Anti-thyroid peroxidase antibody (anti-TPO Ab) titers at the baseline in different genotypes of rs6553010, rs13140012, and rs2119882, and the combined genotype of rs13140012 and rs2119882 in patients with Graves’ disease. Low anti-TPO, low anti-TPO Ab titer (≤1:1600); high anti-TPO, high anti-TPO Ab titer (1:6400~1:25,600 and >1:25,600); the number in the column indicates the frequency of the genotype.

There was no significant difference in the frequency of the AA genotype of rs6553010 between the high-anti-TPO group and that in low-anti-TPO group in patients with GD at the baseline (*p* = 0.083, Fig 2A). In contrast, there were significant associations of anti-TPO Ab titers with rs13140012 and rs2119882 (*p* = 0.003 and 0.003, Fig 2B and 2C, respectively). At the same time, there was also a higher percentage of the AATT genotype with the combination of rs13140012 and rs2119882 in the high anti-TPO group than that in low anti-TPO group in patients with GD (Fig 2D, *p* = 0.004).

### Multivariate logistic regression analysis of SNPs of MTNR1A for susceptibility to GD

Demographic characteristics, such as age, sex, a family history of thyroid disease, and smoking, were first included in the univariate analysis to predict the occurrence of GD. Significant factors, in conjunction with rs6553010, rs13140012, and rs2119882 of MTNR1A and the AATT genotype of rs13140012 and rs2119882, were analyzed by a multivariate logistic regression. Both rs2119882 and the AATT genotype were retained in the model for predicting GD (Table 4; *p* = 0.018 and 0.004, respectively). Interestingly, after adjusting for a family history of thyroid disease and smoking, the AA genotype of rs6553010 and AA genotype of rs13140012 were also selected for the regression model for predicting the development of GD (Table 4; *p* = 0.027 and 0.034, respectively). On the other hand, the AA genotype of rs13140012 was excluded from the regression model for predicting the occurrence of GD in males (*p* = 0.101).

**Table 4:** Multivariate logistic regression analysis to predict the development of Graves’ disease (GD).

	Genetic variant	Smoking	FH
	AOR (95% CI)	AOR (95% CI)	AOR (95% CI)
rs6553010			
AA	1	1.84 (1.20~2.81)*	5.74 (3.50~9.42)*
AG+GG	0.70 (0.50~0.97)*		
rs13140012			
AA	1	1.81 (1.19~2.77)*	5.66 (3.45~9.29)*
AT+TT	0.70 (0.49~0.96)*		
rs2119882			
TT	1	1.79(1.17~2.75)*	5.62(3.43~9.22)*
TC+CC	0.67 (0.48~0.93)*		
rs13140012/rs2119882			
AATT	1	1.81 (1.18~2.76)*	5.76 (3.51~9.47)*
Non AATT	0.61 (0.43~0.85)*		

* *p* < 0.05; AOR, adjusted odds ratio; CI, confidence interval; FH, family history of thyroid disease.

## Discussion

In this report, we studied the role of melatonin-MTNRs in AITD by both SNP analyses of clinical specimens and animal studies. We first demonstrated that a genetic variant of MTNR1A, rs2119882, is associated with susceptibility to GD in an ethnic Chinese (i.e., Taiwanese) population. Interestingly, after adjusting for demographic parameters, the AA genotypes of rs6553010 and rs13140012 and the AATT genotypes of rs13140012 and rs2119882 were also likely to be associated with the development of GD. The AA genotype of rs13140012 and TT genotype of rs2119882 were associated with high anti-TPO titers in GD. Meanwhile, the AATT genotypes of rs13140012 and rs2119882 were also correlated with high anti-TPO titers in GD. This evidence supports the involvement of the melatonin-MTNR signal pathway in the pathogenesis of AITD. Thus, our results suggest that subjects with risk alleles, genotypes, or haplotypes of MTNR1A SNPs should avoid stress and smoking or other risk factors to prevent the occurrence of GD. In addition, healthy subjects and GD patients carrying the risk alleles or genotypes should avoid melatonin administration, which might alter the activity of the melatonin-MTNR1A signal pathway, and subsequently induce the development of GD or thyroid dysfunction. We believe that the findings can offer important information in clinical settings.

Previous studies reported that melatonin acts as a modulator of both cell- and antibody-mediated immunity through membrane-bound MTNRs expressed by immune cells, and alterations of the melatonin-MTNR signal pathway might contribute to the development or different clinical phenotypes of autoimmune diseases [17,18,33]. However, in the past, clinical studies that explored associations of MTNRs with autoimmune diseases were limited and only focused on MTNR1B. Ha et al. showed that rs1562444, an SNP of MTNR1B, was not associated with a risk of rheumatoid arthritis, but was correlated with the presence of a rheumatoid factor in rheumatoid arthritis patients [17]. At the same time, Tanev et al. demonstrated that rs10830963 of MTNR1B was not associated with the development of systemic lupus erythematosus but influenced the risk of leukopenia in systemic lupus erythematosus patients [18]. In animal studies, by inhibiting MTNR1A or MTNR1B gene expression, Drazen et al. suggested that that MTNR1B was likely to be more essential for promoting melatonin-induced cell-mediated and humoral immunity than was MTNR1A in mice [34]. However, evidence showed that the MT1 receptor is much more abundant than the MT2 receptor in the body. Moreover, the MT1 receptor is extensively distributed in the immune system, including the spleen, thymus, and CD4 and CD8 lymphocytes, while the MT2 receptor was only detected in the thymus, which implies that the MT1 receptor may play a more predominant role in immune regulation than does the MT2 receptor [35,36]. At the same time, melatonin can be synthesized by parafollicular C cells of the thyroid gland, and the MT1 receptor was detectable in the thyroid gland, both of which support our results that genetic variants of MTNR1A could influence susceptibility to AITD development [25,37].

In GD, thyroid autoantibodies other than the TSHRAb, including the anti-TPO Ab and ATA, can be found in the bloodstream. Among these, the anti-TPO Ab is the most common type, with a prevalence of about 75%~90% in GD [38,39]. The production of the anti-TPO Ab or ATA in GD may result from overexpression of TPO and TG by thyroid follicular cells, which is driven by the TSHRAb, and subsequently enhances presentation of these autoantigens to immunocytes and finally promotes the formation of the anti-TPO Ab and ATA (Fig B in S1 File) [40,41]. On the other hand, the presence of the anti-TPO Ab and ATA in GD may also imply a concurrence of GD and HT (Fig B in S1 File) [42]. Actually, in addition to genes that participate in the process of antigen-presenting cell-mediated T-cell activation, increasing evidence shows the importance of B-cell activation in regulating susceptibility to AITD [43]. Recent findings in a study of the role of B-cell activating factor in regulating AITD autoantibody production also support this viewpoint. AITD patients had higher serum B-cell-activating factor levels, and its levels were correlated with TSHRAb levels, anti-TPO Ab levels, and ATA titers in female patients, especially those with active GD [44]. Genetic variants of the B-cell-activating factor also affect the occurrences of GD and AITD, and thyroid autoantibody production [28]. Along with the regulatory effects of melatonin on B-cell-activating factor production [29], the melatonin-MTNR pathway may also exert regulatory actions on B cell activation and autoantibody production. In this study, we found no association of MTNR1A SNPs with TSHRAb levels; thus genetic variants only play an important role in the existence of the TSHRAb and development of GD but not the TSHRAb level (Table 1, Fig A in S1 File). On the contrary, we observed that the AA genotypes of rs13140012 and rs2119882 and the combined AATT genotypes of rs13140012 and rs2119882 of MTNR1A were associated with high anti-TPO Ab concentrations in GD. These findings suggest that genetic variants of MTNR1A might influence the diversity of thyroid autoantibody production in GD, which further supports the notion that the melatonin-MTNR pathway may play a role in modulating B-cell functions, i.e., through regulating the B-cell-activating factor. However, the detailed molecular mechanism underlying these findings is unclear, and further study is needed to investigate the mechanism. Finally, there was no significant association of these SNPs with the severity of GD or HT, which was not a surprising result. Evidence has shown that multiple genes and environmental factors participate in the occurrence and modifications of clinical features and treatment outcomes of GD, and the genetic effect of MTNR1A alone might not be strong enough to affect thyroid function. Moreover, in addition to TSHRAb titers, several clinical parameters, including the age at diagnosis, iodine uptake, and thyroid disease history, could also contribute to the discrepancy in initial thyroid function in GD patients [45].

The human MTNR1A gene was first cloned by Reppert et al., and it encodes the MT1 protein containing 350 amino acids [46,47]. rs2119882 is located in the promoter region, the binding site of transcription factors, and may be associated with MTNR1A gene expression and biological functions [48]. It was reported that hypermethylation of the CpG site in the promoter region in which rs2119882 is located is negatively associated with MTNRA messenger (m)RNA expression in oral squamous cell lines [48]. We hypothesized that genetic variants of MTNR1A might modify the function and immunoregulatory activity of MTNR1A and lead to the occurrence of GD. In the present study, we also showed that the AA genotype of both rs13140012 and rs6553010 might also be associated with GD. Both rs13140012 and rs6553010 are located in an intron, which may be associated with splicing of RNA and alterations in MTNR1A mRNA levels. At the same time, it is also possible that rs13140012 is highly associated with the adjacent linked coding region, such as rs2119882 (D' = 0.80), which is associated with the development of GD. Moreover, using electrophoretic mobility shift assays, Esposito et al. suggested that the T allele of the intronic SNP of rs13140012 could enhance the binding affinity of transcription factors compared to the A allele which modifies MTNR1A gene expression [49]. Further in vitro studies to clarify the biological activities of these SNPs are needed.

In summary, to our knowledge, this is the first report to show a possible association of genetic variants of MTNR1A with susceptibility to GD and diversity in the anti-TPO titer. Results of the genetic study suggest that the melatonin-MTNR pathway could play a significant role in the development and modulation of GD. However, we should indicate certain limitations of the study. First, melatonin levels were not measured in the study, and associations of MTNR1A SNPs with serum melatonin remain to be elucidated. It would be valuable to assess associations among genetic variants of MTNR1A, melatonin levels, and clinical severity. Second, the current study is only a cross-sectional study, and the roles of genetic variants of MTNR1A in determining the remission rate after treatment with an anti-thyroid drug or recurrence rate after discontinuation of medication in GD are unclear. Further well-designed longitudinal studies to assess the genetic effects on remission and recurrence rates of GD are required. Finally, as aforementioned, our results strongly imply that the melatonin-MTNR pathway could be involved in the occurrence and modification of clinical features of GD; however, the actual influence of melatonin in GD was not explored in the present study. It is our future plan to analyze alterations in clinical manifestations, including thyroid function, thyroid autoantibody titers, pathology of the thyroid gland, and thyroid-specific gene expressions after administrating melatonin in a well-established GD animal model to further investigate the role of the melatonin-MTNR pathway in regulating GD.

## Supporting information

S1 File Supplementary figures and tables. (PDF)

## Abbreviations

AITD: autoimmune thyroid disease
anti-TPO Ab: anti-thyroid peroxidase antibody
ATA: anti-thyroglobulin antibody
GD: Graves’ disease
HT: Hashimoto’s thyroiditis
MTNR1A: melatonin receptor type 1A
MTNR1B: melatonin receptor type 1B
SNP: single-nucleotide polymorphism
T4: thyroxine
TPO: thyroid peroxide
TSH: thyroid-stimulating hormone
TSHRAb: thyroid-stimulating hormone receptor antibody
