# Supplementary material for: Associations of melatonin receptor gene polymorphisms with Graves' disease
Source: PLoS One. 2017 Sep 29;12(9):e0185529. doi: 10.1371/journal.pone.0185529 (PMC5621676; doi:10.1371/journal.pone.0185529)
Supplement: S1 File — (PDF) [file pone.0185529.s001.pdf]

Table A. Single-nucleotide polymorphisms (SNPs) in melatonin receptor type 1A (MTNR1A) and type 1B (MTNR1B)

SNP	Locus	Location	Base change
rs6553010	186535189	Intron	A/G
rs13140012	186544404	Intron	A/T
rs2119882	186555751	Promoter	C/T
rs1387153	92940662	Downstream	C/G
rs10830963	92975544	Intron	A/G
rs1562444	92982683	3'UTR	C/T

Abbreviation:

UTR, untranslated region.

Table B. Demographic characteristics in Graves' disease, Hashimoto's thyroiditis , autoimmune thyroid disease, and control groups

	Control	GD	HT	AITD
Age (years)	43.3±11.5[c,d]	44.8±12.5	49.8±13.3[a]	45.8±12.9[a]
Gender (female, %)	65.7[c,d]	70.2[c]	90.4[a,b]	74.3[a]
Smoking (%)	14.9[b,d]	23.2[a]	17.3	22.0[a]
Family history of thyroid disease (%)	6.5[b,c,d]	29.6[a]	20.0[a]	27.6[a]

Age is expressed as the mean±standard deviation.

Abbreviation: GD, Graves' disease; HT, Hashimoto's thyroiditis; AITD, autoimmune thyroid disease

[a] $p < 0.05$ vs. the control group, [b] $p < 0.05$ vs. GD, [c] $p < 0.05$ vs. HT, [d] $p < 0.05$ vs. AITD.

Table C. Genotypic and allelic frequencies of rs1387153, rs10830963, and rs1562444 in the melatonin receptor type 1B gene without exclusion of subjects with glucose intolerance

Polymorphism	Control<br>*n* (%)	GD<br>*n* (%)	HT<br>*n* (%)	AITD<br>*n* (%)	OR1 (95% CI)	OR2 (95% CI)	OR3 (95% CI)
rs1387153							
CC	119 (33.1)	96 (30.2)	27 (32.5)	123 (30.7)	1	1	1
CT	165 (46.0)	155 (48.7)	44 (53.0)	199 (49.6)	1.16 (0.82~1.65)	1.18 (0.69~2.01)	1.17 (0.84~1.62)
TT	75 (20.9)	67 (21.1)	12 (14.5)	79 (19.7)	1.11 (0.72~1.69)	0.71 (0.34~1.48)	1.02 (0.68~1.55)
CT+TT	240 (66.9)	222 (69.8)	56 (67.5)	278 (69.3)	1.15 (0.83~1.59)	1.03 (0.62~1.71)	1.12 (0.83~1.52)
Allele							
C	403 (56.1)	347 (54.6)	98 (59.0)	445 (55.5)	1	1	1
T	315 (43.9)	289 (45.4)	68 (41.0)	357 (44.5)	1.07 (0.86~1.32)	0.89 (0.63~1.25)	1.03(0.84~1.26)
rs10830963							
CC	114 (31.8)	96 (30.2)	25 (30.1)	121 (30.2)	1	1	1
CG	178 (49.6)	148 (46.5)	45 (54.2)	193 (48.1)	0.99 (0.70~1.40)	1.15 (0.67~1.98)	1.02 (0.74~1.42)
GG	67 (18.6)	74 (23.3)	13 (15.7)	87 (21.7)	1.31 (0.86~2.01)	0.89 (0.42~1.85)	1.22 (0.81~1.84)
CG+GG	245 (68.2)	222 (69.8)	58 (69.9)	280 (69.9)	1.08 (0.78~1.49)	1.08 (0.64~1.81)	1.08 (0.79~1.46)
Allele							
C	406 (56.5)	340 (53.5)	95 (57.2)	435 (54.2)	1	1	1
G	312 (43.5)	296 (46.5)	71 (42.8)	367 (45.8)	1.13 (0.91~1.40)	0.97 (0.69~1.37)	1.10 (0.90~1.35)
rs1562444							
AA	159 (44.3)	150 (47.2)	39 (47.0)	189 (47.1)	1	1	1
AG	162 (45.1)	134 (42.1)	40 (48.2)	174 (43.4)	0.88 (0.64~1.21)	1.01 (0.62~1.65)	0.90 (0.67~1.22)
GG	38 (10.6)	34 (10.7)	4 (4.8)	38 (9.5)	0.95 (0.57~1.59)	0.43 (0.15~1.27)	0.84 (0.51~1.38)
AG+GG	200 (55.7)	168 (52.8)	44 (53.0)	212 (52.9)	0.89 (0.66~1.21)	0.90 (0.56~1.45)	0.89 (0.67~1.19)
Allele							
A	480 (66.9)	434 (68.2)	118 (71.1)	552 (68.8)	1	1	1
G	238 (33.1)	202 (31.8)	48 (28.9)	250 (31.2)	0.94 (0.75~1.18)	0.82 (0.57~1.19)	0.91 (0.74~1.13)

GD, Graves' disease; HT, Hashimoto's thyroiditis; AITD, autoimmune thyroid disease (GD + HT); control, control group. OR1, odds ratio 1, GD vs. the control; OR2, odds ratio 2, HT vs. the control; OR3, odds ratio 3, AITD vs. the control; CI, confidence interval; * p<0.05.

Table D. Genotypic and allelic frequencies of rs6553010, rs13140012, and rs2119882 in the melatonin receptor type 1A gene in females and males

Polymorphism	Female Control *n* (%)	Female GD *n* (%)	Male Control *n* (%)	Male GD *n* (%)	Female-OR (95% CI)	Male-OR (95% CI)
rs6553010						
AA	113 (47.9)	113 (50.7)	46 (40.0)	49 (51.6)	1	1
AG	95 (40.3)	87 (39.0)	54 (47.0)	33 (34.7)	0.92 (0.62~1.35)	0.57 (0.32~1.04)
GG	28 (11.8)	23 (10.3)	15 (13.0)	13 (13.7)	0.82 (0.45~1.51)	0.81 (0.35~1.89)
AG+GG	123 (52.2)	110 (49.3)	69 (60.0)	46 (48.4)	0.89 (0.62~1.29)	0.63 (0.36~1.08)
Allele						
A	321 (68.0)	313 (70.2)	146 (63.5)	131 (68.9)	1	1
G	151 (32.0)	133 (29.8)	84 (36.5)	59 (31.1)	0.90 (0.68~1.20)	0.78 (0.52~1.18)
rs13140012						
AA	98 (41.5)	104 (46.6)	42 (36.5)	45 (47.4)	1	1
AT	111 (47.0)	94(42.2)	54 (47.0)	42 (44.2)	0.80 (0.54~1.18)	0.73 (0.41~1.30)
TT	27 (11.4)	25 (11.2)	19 (16.5)	8 (8.4)	0.87 (0.47~1.61)	0.39 (0.16~0.99)*
AT+TT	138 (58.4)	119 (53.4)	73 (63.5)	50 (52.6)	0.81 (0.56~1.18)	0.64 (0.37~1.11)
Allele						
A	307 (65.0)	302 (67.7)	138 (60.0)	132 (69.5)	1	1
T	165 (35.0)	144 (32.3)	92 (40.0)	58 (30.5)	0.89 (0.67~1.17)	0.66 (0.44~0.99)*
rs2119882						
TT	97 (41.1)	106 (47.5)	43 (37.4)	47 (49.5)	1	1
CT	111 (47.0)	94 (42.2)	55 (47.8)	38 (40.0)	0.78 (0.53~1.14)	0.63 (0.35~1.13)
CC	28 (11.9)	23 (10.3)	17 (14.8)	10 (10.5)	0.75 (0.41~1.39)	0.54 (0.22~1.30)
CC+CT	139 (58.9)	117 (52.5)	72 (62.6)	48 (50.5)	0.77(0.53~1.11)	0.61 (0.35~1.06)
Allele						
T	305 (64.6)	306 (68.6)	141 (61.3)	132 (69.5)	1	1
C	167(35.4)	140 (31.4)	89 (38.7)	58(30.5)	0.84(0.64~1.10)	0.70 (0.46~1.05)

GD, Graves' disease; control, control group. Female-OR, odds ratio, GD vs. the control in females; Male-OR, odds ratio , GD vs. the control in males; CI, confidence interval; * p<0.05.

Table E. Combined haplotype frequencies of rs6553010, rs13140012 and rs2119882 in Graves' disease (GD) and in controls

rs2119882 C/T	rs13140012 A/T	rs6553010 A/G	GD (318)	Control (351)	OR (95% CI)	*p* value
T	A	A	0.593	0.537	1.26 (1.01~1.56)	0.039
C	T	G	0.195	0.222	0.85 (0.65~1.10)	0.222
C	T	A	0.083	0.089	0.92 (0.67~1.35)	0.674
T	A	G	0.056	0.043	1.30 (0.79~2.74)	0.299
T	T	G	0.034	0.043	0.79 (0.45~1.39)	0.410
Others*			0.039	0.066		

* Others, GAC, AAC, ATT

OR, odds ratio; CI, confidence interval.

Table F. Combined haplotype frequencies of rs1387153, rs10830963 and rs1562444 in Graves' disease (GD) and controls

Total	GD (303)	Control (299)	OR (95% CI)	*p* value
TGA	0.397	0.380	1.07 (0.85~1.36)	0.532
CCG	0.301	0.313	0.94 (0.74~1.20)	0.635
CCA	0.187	0.204	0.90 (0.67~1.19)	0.452
CGA	0.060	0.049	1.25 (0.35~1.03)	0.379
Others*	0.055	0.054		

* Others, TCA, TCG, CGG, TGG.

OR, odds ratio; CI, confidence interval.

Table G. Associations of thyroid function at the baseline with the six single nucleotide polymorphisms in Graves' disease (GD) and Hashimoto's thyroiditis (HT)

	GD		HT	
	Free T4 (ng/dl)	TSH (μU/ml)	Free T4 (ng/dl)	TSH (μU/ml)
rs6553010				
AG+GG	4.41±1.82	<0.01	0.89±0.71	29.09±75.04
AA	4.54±1.87	<0.01	0.88±0.53	15.32±16.62
p value	0.606	-	0.945	0.334
rs13140012				
AT+TT	4.40±1.88	<0.01	0.80±0.53	30.01±71.43
AA	4.56±1.81	<0.01	1.07±0.82	10.42±13.40
p value	0.466	-	0.097	0.188
rs2119882				
CC+CT	4.40±1.86	<0.01	0.84±0.69	30.59±72.74
TT	4.55±1.83	<0.01	0.97±0.54	10.80±13.44
p value	0.529	-	0.461	0.174
rs13140012/rs2119882				
NonAATT	4.45±1.88	<0.01	0.86±0.66	28.12±69.19
AATT	4.51±1.80	<0.01	0.96±0.61	11.90±14.22
p value	0.810	-	0.572	0.303
rs1387153				
CT+TT	4.50±1.84	<0.01	0.87±0.62	27.28±70.9
CC	4.40±1.87	<0.01	0.92±0.70	16.47±23.27
p value	0.699	-	0.769	0.469
rs10830963				
CG+GG	4.56±1.85	<0.01	0.86±0.63	28.98±70.82
CC	4.28±1.82	<0.01	0.95±0.70	12.00±15.59
p value	0.289	-	0.575	0.261
rs1562444				
AG+GG	4.33±1.90	<0.01	0.95±0.75	30.89±79.95
AA	4.63±1.77	<0.01	0.80±0.49	16.53±16.50
p value	0.190	-	0.334	0.268

Free T4 and thyroid-stimulating hormone (TSH) values are expressed as mean ± standard deviation.

Fig A. Thyroid-stimulating hormone receptor antibody (TSHRAb) titers at the baseline in different genotypes of rs6553010, rs13140012, and rs2119882, and genotypes of rs13140012 and rs2119882 in patients with Graves' disease

1

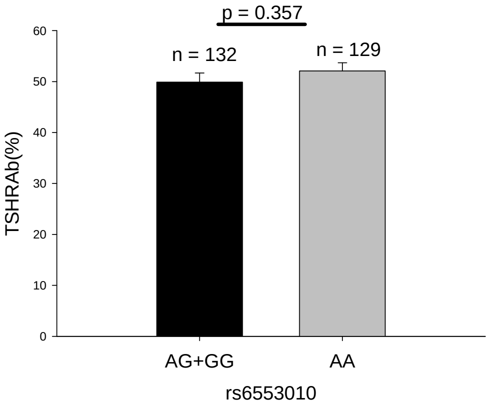

2

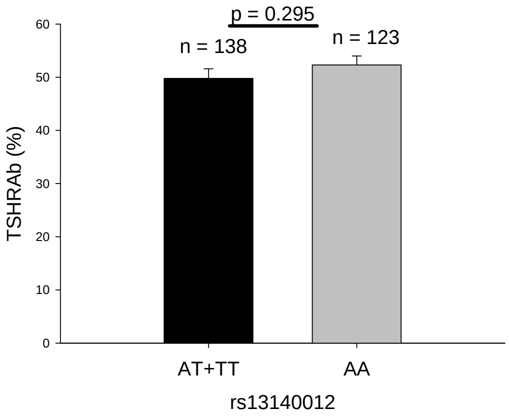

3

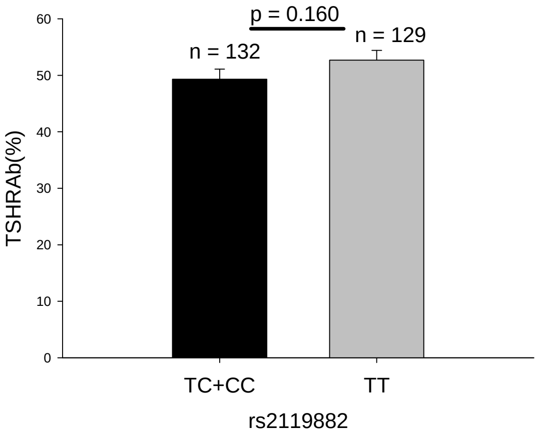

4

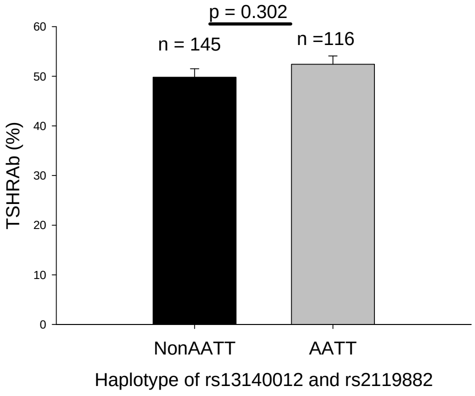

Fig B. The formation of anti-thyroid peroxidase and anti-thyroglobulin antibodes in Graves' disease.

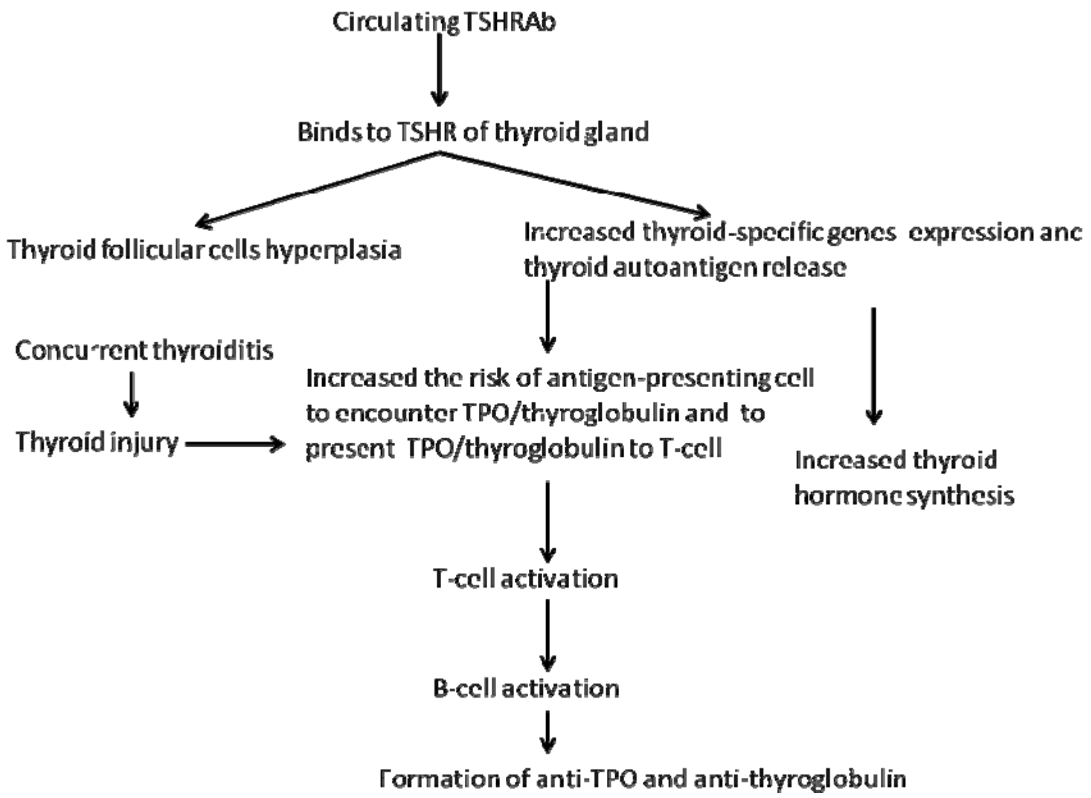

The figure was drawn according to the statement from page 124 in "Autoimmune Disease in Endocrinology", edited by Anthony P. Weetman.
